# Automated macular segmentation can distinguish glaucomatous from compressive optic neuropathy

**DOI:** 10.1007/s00417-022-05962-6

**Published:** 2023-01-10

**Authors:** Ricardo Machado Soares, Joana Figueiredo Braga, Joana da Silva Fernandes, Catarina Cunha Ferreira, Lígia Ribeiro, Hélio Alves, Dália Meira

**Affiliations:** 1 Department of Ophthalmology - Centro Hospitalar de Vila Nova de Gaia e Espinho EPE, Porto, Portugal; 2 Department of Biomedicine - Faculty of Medicine of University of Porto, Porto, Portugal

**Keywords:** Hypothalamic neoplasms, Optic atrophy, Compressive optic neuropathy, Glaucomatous optic neuropathy, Optical coherence tomography

## Abstract

**Purpose:**

To compare macular damage in glaucomatous optic neuropathy (GON) and compressive optic neuropathy (CON) and assess its diagnostic accuracy in distinguishing between diseases.

**Methods:**

Observational, cross-sectional, single-center study. Patients with GON, CON, and healthy controls were included according to the eligibility criteria. An automated spectral-domain optical coherence tomography (SD-OCT) algorithm was used to segment the circumpapilary retinal nerve fiber layer (cpRNFL) and macula. The layer thickness was measured in each sector according to the Early Treatment Diabetic Retinopathy Study and the 6-sector Garway-Heath-based grids. Data was compared across all study groups, and the significance level was set at 0.05.

**Results:**

Seventy-five eyes of 75 participants, 25 with GON, 25 with CON, and 25 healthy controls (CG), were included. Macular thickness was diminished in the ganglion cell complex of GON and CON patients compared to CG (*p*<0.05). The best Garway-Heath-based grid parameters for distinguishing GON and CON were the nasal-inferior (NI) and nasal-superior sectors and the NI/temporal inferior (TI) damage ratios in the macular ganglion cell (mGCL) and inner plexiform (IPL) layers. Moreover, the combination of the NI sector and NI/TI damage ratios in both layers had higher discriminative power (AUC 0.909; 95% CI 0.830–0.988; *p*<0.001) than combining parameters in each layer separately.

**Conclusion:**

Our findings suggest that the evaluation of macular segmented layers damage by SD-OCT may be a helpful add-on tool in the differential diagnosis between GON and CON.

**Supplementary Information:**

The online version contains supplementary material available at 10.1007/s00417-022-05962-6.

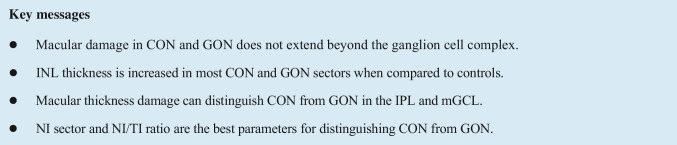

## Introduction

Glaucomatous optic neuropathy (GON) is classically characterized by optic nerve head (ONH) changes (loss of neuroretinal rim and deepening of the optic cup), local or diffuse ganglion cell degeneration, and associated visual field (VF) loss [1–4]. Nevertheless, these hallmark features are not pathognomonic of GON and may overlap with non-glaucomatous neuropathies, leading to misdiagnosis and mismanagement [5–8].

Compressive optic neuropathy (CON) is defined by compression of the optic nerve leading to progressive deterioration of visual acuity. Even though optic disc atrophy is a common presentation of the disease, the retrograde axonal degeneration and nerve fiber loss caused by the compression insult may cause diffuse enlargement of the cup-to-disc ratio (particularly in parasellar or intrasellar tumors), leading to misdiagnosis with GON [8–12]. To avoid the severe consequences of CON misdiagnosis, some studies have proposed neuroradiological imaging of all normal-tension glaucoma (NTG) patients, with very low diagnostic yield, making this approach controversial [12, 13].

Over the years, optical coherence tomography (OCT) has emerged as a valuable noninvasive imaging tool for diagnosing and following optic neuropathies and retinal diseases. Consequently, new parameters based on Bruch’s membrane opening minimum rim width (BMO-MRW) and circumpapillary retinal nerve fiber layer (cpRNFL) have been described to assist in the differential diagnosis of CON [14–18]. Most recently, macular analysis of the ganglion cell complex (GCC) through OCT proved significant in diagnosing GON [19–21]. However, few studies depicting macular damage and using it as a parameter to distinguish between GON and CON exist in the literature. In light of this, the purpose of this study was to compare macular damage in GON and CON and assess its diagnostic accuracy in distinguishing between diseases.

## Materials and methods

### Study design and diagnostic criteria

Cross-sectional, observational, and single-center study was conducted at the neuro-ophthalmology and glaucoma divisions of the Department of Ophthalmology of Centro Hospitalar Vila Nova de Gaia e Espinho. Patients with open-angle GON (ICD11- glaucomatous optic neuropathy: 9C40.9) and CON (ICD11-compressive optic neuropathy: 9C40.5) due to sellar and parasellar tumors and a healthy control group (CG) (selected from healthy subjects who attended the center for a routine ophthalmology examination) were included. Each CON patient in the study group was matched by sex and age (with no more than 3 years of age difference) to an individual in the GON and CG groups. All participants provided written informed consent. The study was approved by the Centro Hospitalar Vila Nova de Gaia e Espinho ethics committee and complied with the tenets of the Declaration of Helsinki for biomedical research.

### Inclusion and exclusion criteria

Inclusion criteria for GON patients comprised:

1. Diagnosis of chronic open-angle glaucoma by a glaucoma specialist requiring topical medication to control the disease progression; AND
2. No previous glaucoma surgery; AND
3. Glaucomatous VF defect (as reported on “glaucoma hemifield test” – at least two exams with a result “outside of normal limits” accompanied by a glaucomatous lesion pattern); AND
4. Structural glaucomatous lesion detected on ophthalmological examination (for example, vertical enlargement of cup/disc ratio; notching of neuroretinal rim; optic disc hemorrhages; bayoneting or baring of circumlinear vessels); AND
5. Structural optic disc defect detected on spectral-domain OCT (SD-OCT) (documented cpRNFL defect in at least two exams).

Patients enrolled in the CON group were selected according to the following criteria:

1. Optic disc atrophy observed on fundoscopy; AND
2. Direct compression of the anterior optic pathway by a space-occupying lesion (SOL) documented on magnetic resonance imaging (at least one year after surgery in case of SOL resection); AND
3. VF defect secondary to SOL, confirmed by a neuro-ophthalmology specialist; AND
4. Structural cpRNFL loss documented on SD-OCT in at least two exams; AND
5. No previous history of GON diagnosis; AND
6. Intraocular pressure (IOP) within normal limits (<21 mmHg).

Exclusion criteria were:

1. Spherical equivalent (SE) >3 diopters or an astigmatism >3 diopters; OR
2. Axial length <22 mm or >25 mm; OR
3. History of intraocular surgery in the previous six months; OR
4. Other ophthalmological pathologies (for example, retinal diseases like an epiretinal membrane and diabetic retinopathy).

According to the eligibility criteria, only one eye of each participant was included in the study. If both eyes met the inclusion criteria, one eye was randomly selected.

### Ophthalmologic examination and data collection

All participants enrolled underwent a complete ophthalmological examination, including best-corrected visual acuity, slit-lamp biomicroscopy, gonioscopy, Goldmann applanation tonometry, and dilated stereoscopic examination of the optic disc. Data collected included base demographics (age, sex) and SE. VF testing was performed using the automated VF analyzer (Humphrey Field Analyzer; Carl Zeiss Meditec, Inc., Dublin, CA, USA). Peripapillary and macula scanning were performed using the SD-OCT (Spectralis®, Heidelberg Engineering GmbH, Heidelberg, Germany). The sector macular thickness of retina layers was collected.

### Visual field testing

GON and CON patients underwent VF automated perimetry using the Swedish Interactive Threshold Algorithm standard strategy, a 24–2 pattern, and a size III white stimulus. VF exam reliability was defined as <20% fixation loss rate, <15% false-positive rate, and <15% false-negative rate. The mean deviation (MD) results were collected, and non-reliable test results were not included for the analysis. For descriptive purposes, glaucoma patients were subsequently stratified according to their MD using the simplified version of the *Hodapp-Anderson-Parrish* glaucoma staging system as mild glaucoma [MD ≥-6.00 dB], moderate glaucoma (MD between −6.01 and −12.00 dB) and severe glaucoma (MD <−12.00 dB) [22, 23].

### Spectral-domain optical coherence tomography

The cpRNFL and macular analyses were performed using the SD-OCT Glaucoma Module Premium Edition software (*Spectralis*®, Heidelberg Engineering GmbH, Heidelberg, Germany), which uses the Anatomic Positioning System (APS) to align the image according to two fixed landmarks — the center of the BMO and the center of the fovea.

The cpRNFL thickness was assessed using a 12° circular scan (3,5 mm diameter) centered on the BMO, and the results obtained were collected in a 6-sector Garway-Heath-based grid [24] — nasal-superior (NS, 90–135°), nasal (N, 135–225°), nasal-inferior (NI, 225–270°), temporal-inferior (TI, 270–315°), temporal (T, 315–45°), and temporal-superior (TS, 45–90°). Global and sectorial values were collected from each patient.

**Fig. 1:**
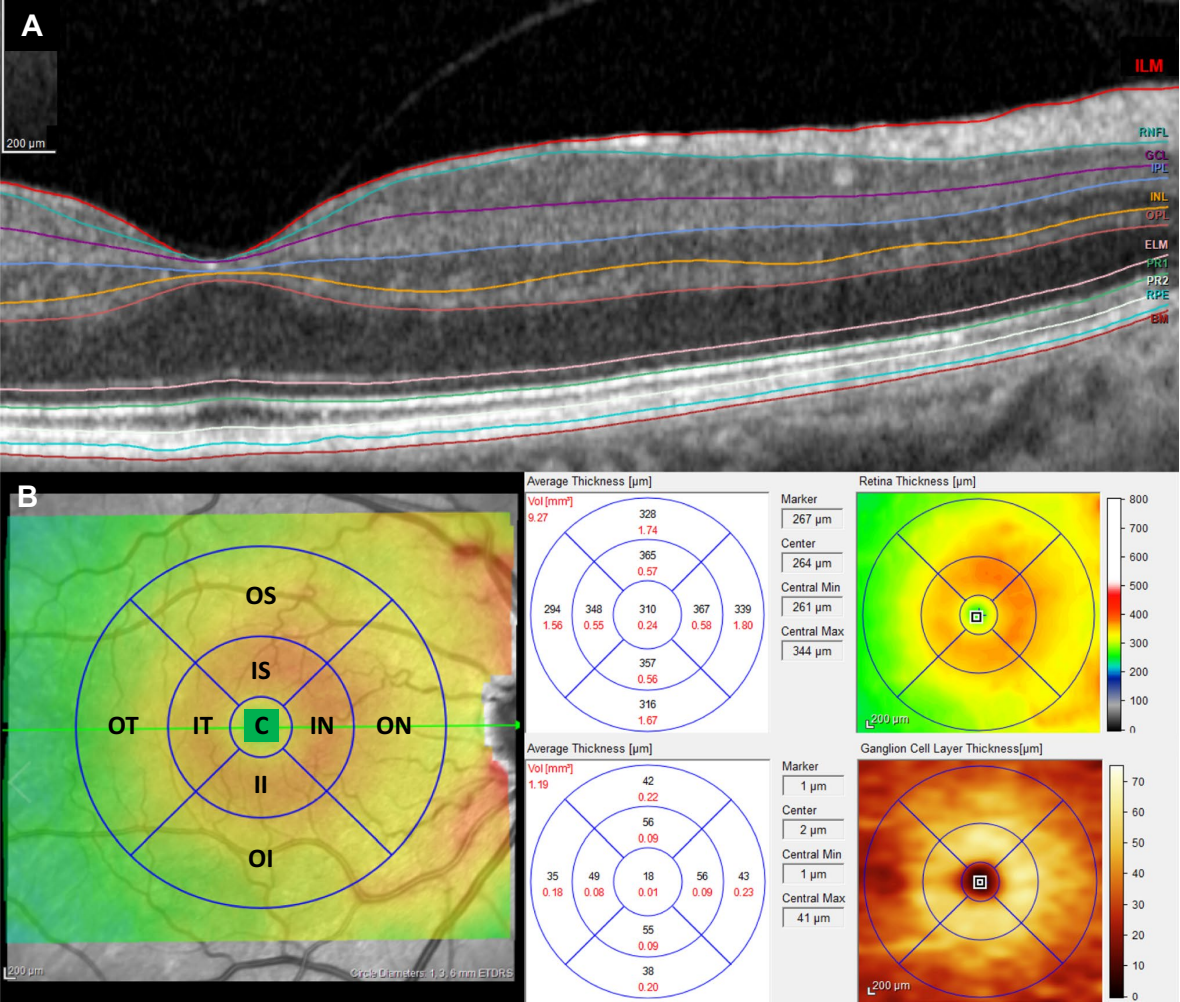
Automated macular segmentation performed by the SD-OCT and ETDRS grid. **A** macular segmentation of every retinal layer; **B** macular division according to the ETDRS grid in the macular ganglion cell layer. ETDRS sectors: C, central; II, inner inferior; IN, inner nasal; IS, inner superior; IT, inner temporal; OI, outer inferior; ON, outer nasal; OS, outer superior; OT, outer temporal

**Fig. 2:**
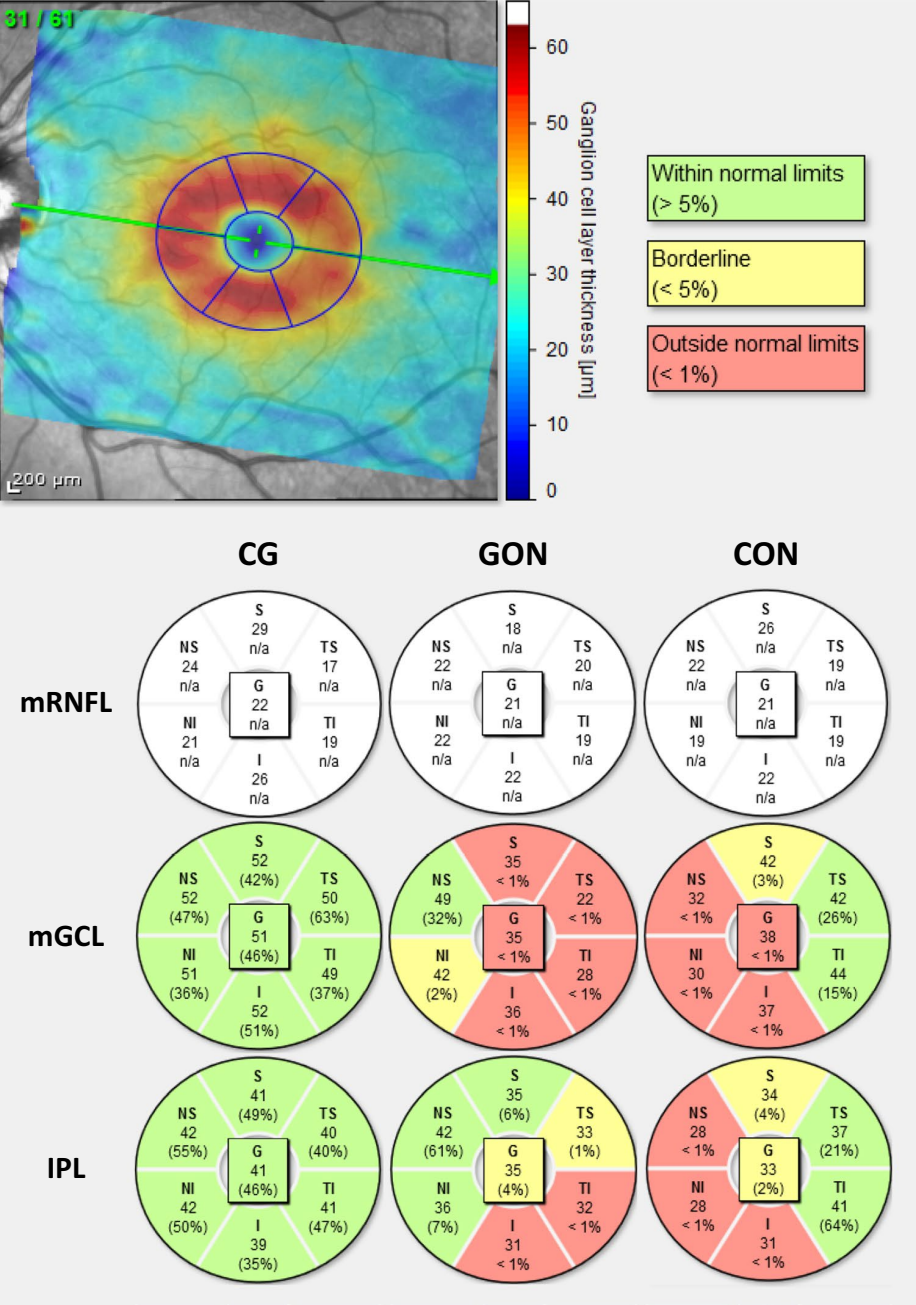
Macular division according to the Garway-Heath-based grid. Sectors are colored in green (within normal limits), yellow (borderline), and red (outside normal limits) according to the device’s reference database. CG, healthy control group; CON, compressive optic neuropathy; GON, glaucomatous optic neuropathy; IPL, inner plexiform layer; mGCL, macular ganglion cell layer; mRNFL, macular retinal nerve fiber layer. Garway-heath grid sectors: G, Global; I, Inferior; NI, nasal-inferior; NS, nasal-superior; S, superior; TI, temporal-inferior; TS, temporal-superior

Macular thickness measurements were obtained using the Glaucoma Module Premium Posterior Pole scan. This software uses 61 B-scans (each B-scan consists of 768 A-scans) spaced 120 μm apart (30°×25° volume scan) to segment the retina into eight layers — the inner retinal (measured from the internal limiting membrane to the external limiting membrane layers) and outer retinal (measured from the external limiting membrane layer to the Bruch’s membrane) layers (Fig. 1A). Subsequently, the macula of each layer was divided according to two classifications — Early Treatment Diabetic Retinopathy Study (ETDRS) grid and the 6-sector Garway-Heath-based grid. The ETDRS grid divided the macula of all retina layers according to the fovea localization into inner and outer (3- and 6-mm diameter, respectively) rings. The average of all points within the inner 1-mm circle radius was defined as central (C) thickness. The inner ring was further divided into — inner superior (IS), inner nasal (IN), inner inferior (II), and inner temporal (IT) sectors, and the outer ring into outer superior (OS), outer nasal (ON), outer inferior (OI), and outer temporal (OT) sectors (Fig. 1B). Numerical values recorded in each of the nine ETDRS subfields were collected. Unlike the ETDRS grid, the 6-sector Garway-Heath*-*based grid classification uses the APS software to align the macula according to the BMO-fovea axis, segmenting the macula of the three GCC layers [retinal nerve fiber layer (mRNFL), ganglion cell layer (mGCL), and inner plexiform layer (IPL)] into six sectors: superior (S), TS, TI, inferior (I), NI, and NS sectors (Fig. 2). Global and sector values were collected from every patient. All images were reviewed for disc centration, foveal fixation, segmentation errors, and image artifacts. Minor segmentation errors were manually corrected with the device’s built-in software. The analysis did not include SD-OCT scans with major segmentation errors, poor centering, or quality (signal strength <20 dB). Macular thickness collection was performed by the two investigators to guarantee the repeatability/agreement of the data.

### Statistical analysis

Statistical analysis was conducted using SPSS statistical software (IBM SPSS statistics package version 28, NY, USA). For descriptive purposes of the study sample, counts and proportions were presented. The Shapiro-Wilk test was performed to evaluate the normality of the distributions. Symmetrical variables were described using means ± standard deviation, and non-symmetrical distributions were described using the median and interquartile range. Comparisons between independent variables (age, sector cpRNFL, and sector macular thickness) were performed using ANOVA and Kruskal-Wallis tests, followed by pairwise comparisons for each duo (Bonferroni and Dunn’s tests, respectively) whenever a significant result was obtained. VF MD in GON and CON patients were compared using the Mann-Whitney *U* test. The resulting *p*-values were adjusted using Bonferroni and Dunn corrections based on the number of comparisons in each analysis and the significance level was set at *α*=0.05. The discriminative power for differentiating GON and CON in the mRNFL, mGCL, and IPL sectors (6-sector Garway-Heath classifications) was assessed by constructing receiver operating characteristic (ROC) curves and analyzing their area under the ROC curve (AUC). Logistic regression was performed to combine different parameters, followed by AUC analysis to ascertain the discriminative power of parameter combination. Youden’s index was used to calculate the AUC’s optimal measure value (Ĵ) for the best-performing macular sectors and nasal-temporal ratios in the Garway-Heath-based grid.

## Results

### Demographic and clinical data

A total of 75 eyes of 75 patients (37 male and 38 female) were included in the study (25 per group) and the mean age of the sample was 61.24 ± 11.41 years (range, 29–86). No statistically significant differences were found between groups regarding sex and age (*p*>0.05). Concerning clinical data, the mean SE of patients was 0.13 ± 1.03 in the CG, 0.08 ± 0.72 in GON, and 0.38 ± 0.94 in CON (*p*=0.460). Median VF MD was −6.01 (11.65) dB in GON patients and −7.55 (12.85) dB in CON patients (*p*=0.552). When staging GON patients, 48% had mild glaucoma (*n*=12), 24% moderate glaucoma (*n*=6), and 28% severe glaucoma (*n*=7). In the CON group, 56% of patients had a pituitary macroadenoma (*n*=14), 36% had meningiomas (*n*=9) and 8% of patients had craniopharyngioma (*n*=2). The demographical and clinical information of each group at the time of examination is presented in Table 1.

**Table 1:** Demographic and clinical characteristics of the groups

	CG		GON		CON		CG x GON x CON Adj. *p***-**value
Number of patients (%)	25	(33.3)	25	(33.3)	25	(33.3)	
Age, years (mean ± SD)	60.12	(9.48)	61.72	(12.22)	61.88	(12.65)	0.837
Sex (number, %)							
Male	12	(48.0)	16	(64.0)	9	(36.0)	0.139
Female	13	(52.0)	9	(36.0)	16	(64.0)	
Spherical equivalent, D (mean ± SD)	0.13	(1.03)	0.08	(0.72)	0.38	(0.94)	0.460
Visual field MD, dB [median (IQR)]			−6.01	(11.65)	−7.55	(12.85)	0.552
Glaucoma staging (number, %)							
Mild			12	(48)			
Moderate			6	(24)			
Severe			7	(28)			
Space occupying lesion (number, %)							
Pituitary macroadenoma					14	(56)	
Meningioma					9	(36)	
Craniopharyngioma					2	(8)	

*Adj*, adjusted; *CON*, compressive optic neuropathy; *CG*, healthy control group; *D*, diopters; *dB*, decibels; *IQR*, interquartile range; *MD*, mean deviation; *SD*, standard deviation

### Spectral-domain optical coherence tomography parameters

#### Circumpapillary retinal nerve fiber layer

A detailed description and analysis (including AUC) of cpRNFL measurements in each group are reported in Table 2. Compared to the CG, cpRNFL was significantly thinner in every sector in both GON and CON (*p*<0.001). No significant differences were observed between GON and CON (*p*>0.05).

#### ETDRS grid macular thickness analysis

Description and analysis of ETDRS subfield macular thickness values in the mRNFL, mGCL, IPL, and INL of each group are summarized in Table 2. In comparison to the CG group, GON’s macular thickness was significantly diminished in most subfields of the three GCC retinal layers (*p*<0.05) except in the mRNFL-IN, -IT, and -OT subfields (*p*>0.05). Similarly, when comparing CG with CON patients, most ETDRS subfields were significantly thinner in these three inner retinal layers in CON patients (*p*<0.05) except for mRNFL-IT (*p*=0.368). The inner nuclear layer (INL) was the only layer thicker in GON and CON in almost every sector (except INL-C in GON and INL-C and -IT in CON) compared to the CG, but significant differences were only present in the INL-II (*p*=0.04), -OI (*p*=0.005), and -OT (*p*=0.023) sectors of GON patients. No significant differences in macular thickness were found between groups in the outer plexiform, outer nuclear, outer retinal, and retinal pigment epithelium layers (*p*>0.05). When comparing GON and CON, the IN subfield was consistently thinner in patients with CON in the mRNFL (*p*=0.012), mGCL (*p*=0.013), and IPL (*p*=0.002). The ON subfield was significantly diminished in CON in the mRNFL (*p*=0.041) and IPL (*p*=0.015), and the IS subfield was thinner in CON in the IPL (*p*=0.043). An extensive description of macular thickness measurements in all retina layers is depicted in Supplementary Table 1.

**Table 2:** Circumpapillary RNFL sector thickness (Garway-Heath-based grid) and macular thickness of the GCC layers and INL (ETDRS grid subfields)

Parameters	CG** (***N***=**25)		GON (*N***=**25)		CON (*N***=**25)		CG x GON Adj. *p***-**value	CG x CON Adj. *p***-**value	GON x CON Adj. *p***-**value
cpRNFL									
G	99.00	(9.20)	63.72	(15.28)	61.92	(16.82)	**<0.001**	**<0.001**	1.000
NS	117.40	(20.44)	75.52	(21.13)	71.96	(28.30)	**<0.001**	**<0.001**	0.530
N	83.64	(11.26)	55.96	(15.48)	50.00	(18.68)	**<0.001**	**<0.001**	1.000
NI	125.16	(26.79)	74.00	(26.77)	70.88	(30.01)	**<0.001**	**<0.001**	1.000
TI	148.12	(18.83)	85.92	(36.97)	99.28	(34.49)	**<0.001**	**<0.001**	0.401
T	65.52	(11.11)	47.68	(12.46)	41.76	(13.24)	**<0.001**	**<0.001**	0.280
TS	124.44	(25.24)	76.28	(29.21)	84.20	(29.68)	**<0.001**	**<0.001**	0.968
mRNFL									
C	12.76	(2.45)	10.84	(3.00)	9.76	(2.35)	**0.034**	**<0.001**	0.446
IS	24.24	(2.88)	20.24	(2.88)	18.44	(2.53)	**<0.001**	**<0.001**	0.073
OS*	36.00	(3.00)	28.00	(14.00)	25.00	(9.00)	**<0.001**	**<0.001**	1.000
IN*	21.00	(3.00)	19.00	(4.00)	17.00	(4.00)	0.067	**<0.001**	**0.012**
ON	46.12	(6.15)	32.28	(8.11)	27.16	(7.06)	**<0.001**	**<0.001**	**0.041**
II*	25.00	(3.00)	21.00	(6.00)	18.00	(3.00)	**<0.001**	**<0.001**	0.415
OI	39.44	(5.96)	24.60	(6.93)	26.76	(6.89)	**<0.001**	**<0.001**	0.755
IT*	17.00	(1.00)	18.00	(2.00)	18.00	(4.00)	1.000	0.368	1.000
OT	19.08	(1.68)	18.44	(2.18)	17.36	(1.98)	0.754	**0.008**	0.165
mGCL									
C*	14.00	(7.00)	12.00	(4.00)	9.00	(3.00)	**0.026**	**<0.001**	0.090
IS	51.36	(5.31)	38.12	(11.29)	32.72	(9.00)	**<0.001**	**<0.001**	0.105
OS	34.88	(3.27)	28.00	(5.45)	26.64	(5.38)	**<0.001**	**<0.001**	0.961
IN*	50.00	(7.00)	40.00	(18.00)	23.00	(15.00)	**<0.001**	**<0.001**	**0.013**
ON*	39.00	(7.00)	33.00	(10.00)	26.00	(8.00)	**0.003**	**<0.001**	0.074
II	51.64	(5.41)	36.20	(10.43)	33.28	(10.30)	**<0.001**	**<0.001**	0.768
OI*	33.00	(7.00)	25.00	(7.00)	26.00	(8.00)	**<0.001**	**<0.001**	1.000
IT	48.24	(5.30)	31.48	(9.82)	30.76	(9.50)	**<0.001**	**<0.001**	1.000
OT	35.16	(3.18)	24.76	(5.11)	25.52	(6.77)	**<0.001**	**<0.001**	1.000
IPL									
C*	20.00	(5.00)	18.00	(5.00)	16.00	(3.00)	**0.034**	**<0.001**	0.151
IS*	40.00	(6.00)	37.00	(10.00)	29.00	(7.00)	**0.002**	**<0.001**	**0.043**
OS	28.64	(2.56)	24.96	(3.39)	23.40	(2.89)	**<0.001**	**<0.001**	0.201
IN*	42.00	(6.00)	37.00	(11.00)	26.00	(9.00)	**0.007**	**<0.001**	**0.002**
ON*	30.00	(5.00)	27.00	(7.00)	22.00	(7.00)	**0.04**	**<0.001**	**0.015**
II	40.80	(3.25)	32.68	(6.47)	29.80	(5.58)	**<0.001**	**<0.001**	0.173
OI	27.12	(2.35)	23.40	(3.79)	22.76	(2.80)	**<0.001**	**<0.001**	1.000
IT	42.08	(2.89)	32.92	(6.16)	31.16	(6.30)	**<0.001**	**<0.001**	0.746
OT	32.32	(2.45)	27.20	(3.18)	26.56	(4.14)	**<0.001**	**<0.001**	1.000
INL									
C*	22.00	(10.00)	20.00	(8.00)	18.00	(8.00)	1.000	0.260	0.469
IS	42.80	(4.04)	45.04	(5.80)	45.32	(6.87)	0.505	0.365	1.000
OS	32.40	(2.20)	34.12	(3.18)	33.32	(4.15)	0.202	0.972	1.000
IN*	42.00	(5.00)	45.00	(6.00)	43.00	(7.00)	0.177	0.852	1.000
ON	34.68	(2.67)	36.68	(3.81)	36.48	(4.82)	0.215	0.313	1.000
II*	41.00	(5.00)	45.00	(9.00)	43.00	(9.00)	**0.040**	1.000	0.378
OI*	32.00	(3.00)	34.00	(4.00)	33.00	(5.00)	**0.005**	0.285	0.438
IT	39.68	(3.42)	41.12	(5.97)	39.12	(4.39)	0.851	1.000	0.413
OT*	33.00	(2.00)	35.00	(4.00)	34.00	(5.00)	**0.023**	1.000	0.165

Measurements are presented in μm and are described as mean ± standard deviation or median and interquartile range. *Adj*, adjusted; *CG*, healthy control group; *CON*, compressive optic neuropathy; *cpRNFL*, circumpapillary retinal nerve fiber layer; *ETDRS*, Early Treatment of Diabetic Retinopathy Study; *GCC*, ganglion cell complex; *GON*, glaucomatous optic neuropathy; *IPL*, inner plexiform layer; *INL*, inner nuclear layer; *mGCL*, macular ganglion cell layer; *mRNFL*, macular retinal nerve fiber layer; Garway-Heath-based sectors: *G*, Global; N, nasal; *NI*, nasal-inferior; *NS*, nasal-superior; *T*, temporal; *TI*, temporal-inferior; *TS*, temporal-superior. *ETDRS* subfields: *C*, central; *II*, inner inferior; *IN*, inner nasal; *IS*, inner superior; *IT*, inner temporal; *OI*, outer inferior; *ON*, outer nasal; *OS*, outer superior; *OT*, outer temporal

*Values presented in median and interquartile range

Significant *p*-values are in bold

#### Garway-Heath-based grid macular thickness analysis

Detailed description and analysis (including AUC) of global and sector macular thickness values and nasal/temporal ratios in the GCC layers using the Garway-Heath-based grid are summarized in Table 3. When compared with the CG, the macular thickness was diffusely diminished in the three GCC layers of GON and CON patients (p<0.05) except for the mRNFL-TS sector (*p*>0.05). Regarding GON and CON differences, the NS and NI sectors were significantly thinner in CON in the mGCL (*p*<0.001) and IPL (*p*=0.002) (Fig. 2). Additionally, the global and S sector values were thinner in the IPL of CON patients (*p*=0.002 and *p*=0.008, respectively). In the mRNFL, only the global and NS were diminished in CON (*p*=0.046 and *p*=0.028, respectively). The only diminished sector in GON patients (compared to CON) was the mGCL-TI sector, although this difference was not statistically significant.

**Table 3:** Macular GCC layer sector thickness according to Garway-Heath-based grid

Parameters	CG **(***N***=**25)		GON (*N***=**25)		CON (*N***=**25)		CG x GON Adj. *p***-**value	CG x CON Adj. *p***-**value	GON x CON Adj. *p***-**value	CON x GON		
										AUC	95% CI	Adj*. p-value*
mRNFL												
G	23.82	(3.02)	20.24	(1.94)	18.64	(1.78)	**<0.001**	**<0.001**	**0.046**	0.734	0.596–0.873	**0.004**
S*	26.00	(3.00)	21.00	(5.00)	19.00	(4.00)	**<0.001**	**<0.001**	0.404	0.640	0.484–0.796	0.090
I*	28.00	(4.00)	22.00	(8.00)	20.00	(4.00)	**<0.001**	**<0.001**	1.000	0.568	0.401–0.735	0.410
NS	26.59	(3.92)	21.60	(3.15)	19.12	(2.74)	**<0.001**	**<0.001**	**0.028**	0.710	0.567–0.853	**0.011**
NI*	25.00	(4.00)	21.00	(5.00)	18.00	(2.00)	**0.002**	**<0.001**	0.112	0.698	0.548–0.848	**0.017**
TS*	18.00	(1.00)	18.00	(3.00)	18.00	(3.00)	0.670	1.000	0.560	0.608	0.451–0.765	0.190
TI*	21.00	(4.00)	19.00	(2.00)	18.00	(2.00)	**0.003**	**<0.001**	1.000	0.586	0.428–0.745	0.295
NS/TI	1.26	(0.13)	1.18	(0.24)	1.08	(0.16)	0.468	**0.003**	0.127	0.632	0.476–0.788	0.109
NI/TI	1.16	(0.09)	1.12	(0.18)	1.04	(0.13)	0.768	**0.008**	0.131	0.683	0.532–0.834	**0.026**
mGCL												
G	50.27	(4.85)	36.12	(8.44)	31.08	(7.99)	**<0.001**	**<0.001**	0.054	0.685	0.535–0.834	**0.025**
S	51.14	(5.21)	37.64	(11.16)	33.04	(8.27)	**<0.001**	**<0.001**	0.196	0.622	0.462–0.783	0.138
I	51.05	(5.26)	36.04	(9.50)	31.92	(8.64)	**<0.001**	**<0.001**	0.231	0.636	0.479–0.793	0.099
NS	50.91	(5.30)	40.08	(10.76)	29.56	(8.58)	**<0.001**	**<0.001**	**<0.001**	0.772	0.640–0.904	**0.001**
NI	51.18	(5.11)	38.72	(9.25)	28.00	(8.97)	**<0.001**	**<0.001**	**<0.001**	0.793	0.668–0.917	**<0.001**
TS	47.68	(5.08)	32.08	(10.42)	29.84	(8.30)	**<0.001**	**<0.001**	1.000	0.554	0.390–0.717	0.516
TI	50.23	(5.15)	32.56	(11.35)	34.08	(10.36)	**<0.001**	**<0.001**	1.000	0.466	0.303–0.628	0.677
NS/TI*	1.02	(0.06)	1.13	(0.65)	0.88	(0.35)	0.685	0.103	**0.002**	0.759	0.627–0.891	**0.002**
NI/TI*	1.02	(0.06)	1.13	(0.54)	0.86	(0.36)	0.563	**0.011**	**<0.001**	0.823	0.709–0.937	**<0.001**
NI + NI/TI										0.860	0.759–0.961	**<0.001**
IPL												
G	41.14	(3.17)	33.48	(5.03)	29.04	(4.76)	**<0.001**	**<0.001**	**0.002**	0.736	0.596–0.876	**0.004**
S	40.68	(3.76)	33.64	(6.36)	29.28	(4.11)	**<0.001**	**<0.001**	**0.008**	0.711	0.563–0.860	**0.010**
I*	39.00	(5.00)	34.00	(10.00)	28.00	(7.00)	**<0.001**	**<0.001**	0.191	0.685	0.530–0.839	**0.025**
NS*	41.50	(7.00)	37.00	(12.00)	27.00	(7.00)	**0.018**	**<0.001**	**0.002**	0.802	0.680–0.923	**<0.001**
NI*	42.00	(6.00)	36.00	(10.00)	25.00	(8.00)	**0.003**	**<0.001**	**0.002**	0.843	0.733–0.954	**<0.001**
TS	41.32	(3.12)	32.64	(6.75)	30.52	(5.90)	**<0.001**	**<0.001**	0.547	0.595	0.435–0.755	0.248
TI	40.86	(2.42)	32.00	(6.90)	31.56	(6.14)	**<0.001**	**<0.001**	1.000	0.526	0.364–0.689	0.749
NS/TI*	1.00	(0.08)	0.98	(0.34)	0.95	(0.33)	1.000	0.162	0.087	0.675	0.525–0.825	**0.034**
NI/TI*	1.02	(0.06)	1.06	(0.22)	0.92	(0.32)	1.000	**0.004**	**<0.001**	0.819	0.705–0.933	**<0.001**
NI + NI/TI										0.874	0.777–0.972	**<0.001**
IPL + GCL												
NI + NI/TI										0.909	0.830–0.988	**<0.001**

Measurements are presented in μm and are described as mean ± standard deviation or median and interquartile range. *Adj*, adjusted; *AUC*, area under the curve; *CON*, compressive optic neuropathy; *CI*, confidence interval; *CG*, healthy control group; *GCC*, ganglion cell complex; *G*, global; *I*, inferior; *IPL*, inner plexiform layer; *mGCL*, macular ganglion cell layer; *mRNFL*, macular retinal nerve fiber layer; *NI*, nasal-inferior. *NI/TI*, nasal-inferior/temporal-inferior ratio; NS, nasal-superior; *NS/TI*, nasal-superior/temporal-inferior ratio; *S*, superior; *TI*, temporal-inferior; *TS*, temporal-superior

*Values presented in median and interquartile range

Significant *p*-values are in bold

The anatomical findings of the sectorial analysis — atrophy of the NS and NI sectors in CON compared to atrophy of TI sector in GON — led us to calculate the NS/TI and NI/TI ratios for all GCC layers. In the mRNFL NS/TI and NI/TI ratios were significantly diminished (*p*=0.003 and *p*=0.008, respectively) in CON patients when compared to CG, but no difference was found between GON and CON (p>0.05). The NI/TI ratios in both mGCL and IPL were diminished in CON compared to CG (*p*=0.011 and *p*=0.004, respectively) and GON (*p*<0.001 in both layers). The NS/TI ratio was significantly diminished in CON compared to GON in the mGCL (*p*=0.002), but no differences were found between groups in the IPL (*p*>0.05).

The usefulness of discriminating between CON and GON for each parameter of the GCC sectors (using Garway-Heath grid) and nasal/TI ratios was assessed using the AUC. In the mGCL the NI sector [*Ĵ* = 35.5 (sensitivity=0.84, specificity=0.64); AUC 0.793; 95% CI 0.668–0.917; *p*<0.001] and NI/TI ratio [*Ĵ* = 0,9583 (sensitivity=0.68, specificity=0.88); AUC 0.823; 95% CI 0.709–0.937; *p*<0.001] were the best parameters followed by the NS sector [*Ĵ* = 44 (sensitivity= 0.96, specificity= 0.52); AUC 0.772; 95% CI 0.640–0.904; *p*=0.001]. Likewise, in IPL the NI sector [*Ĵ*= 28.5 (sensitivity=0.72, specificity=0. 88); AUC 0.843; 95% CI 0.733–0.954; *p*<0.001] and NI/TI ratio [*Ĵ* = 0,9648 (sensitivity=0.76, specificity=0.76); AUC 0.819; 95% CI 0.705–0.933; *p*<0.001] were the the best-performing parameters for distinguishing between the two neuropathies, followed by the NS sector [*Ĵ* = 36.5 (sensitivity= 0.96, specificity= 0.52); AUC 0.802; 95% CI 0.680–0.923; *p*<0.001]. The combination of the NI sector damage and NI/TI ratio parameters had higher discriminative power in mGCL (sensitivity=0.68, specificity=0.92; AUC 0.860; 95% CI 0.759–0.961; *p*<0.001) and in IPL (sensitivity=0.88, specificity=0.76; AUC 0.874; 95% CI 0.777–0.972; *p*<0.001) than testing each parameter individually. Finally, the combination of these two parameters in both layers (NI damage and NI/TI ratio in mGCL and IPL) had the highest yield of accuracy in distinguishing between CON and GON (sensitivity=0.84, specificity=0.84; AUC 0.909; 95% CI 0.830–0.988; *p*<0.001).

## Discussion

Distinguishing GON from other non-glaucomatous neuropathies can be challenging. Some studies have highlighted that even when provided with color fundus photographs and automated perimetry exams, glaucoma specialists may misdiagnose non-glaucomatous optic neuropathies in 20–25% of patients [13, 25]. CON is known to mimic the glaucomatous ONH cupping not only on fundoscopy examination but also in quantitative OCT, making it one of the most commonly missed diagnoses in favor of GON [14–17]. Therefore, finding feasible criteria to implement in routine glaucoma care is paramount to identify patients that would benefit from neuroimaging. In this study, we used the SD-OCT segmentation tool to evaluate the cpRNFL and macular thickness of healthy CG and patients with GON and CON. Our results suggest that macular analysis of the NS and NI sectors in conjunction with the NS/TI and NI/TI ratios in mGCL and IPL may be superior to cpRNFL analysis in predicting CON over GON, thus identifying patients warranting further imaging investigation.

Previous studies mentioned the analysis of cpRNFL evaluation for GON and CON differential diagnosis. The rationale behind this analysis arises from the distinct mechanisms of GON, where the axonal injury of the lamina cribrosa of the ONH leads to damage to the more susceptible superior and inferior arcuate fibers (less connective tissue support) compared to CON, where the retrograde axonal degeneration of the chiasmal crossing fibers leads to band atrophy with damage of the nasal hemiretina fibers [26–28]. Danesh-Meyer et al. suggested that cpRNFL sector evaluation using OCT could help differentiate between CON and GON. The authors stated that CON’s cpRNFL thickness was significantly thinner in the nasal and temporal sectors (particularly the 3 o’clock temporal sector) of ONH compared with GON (which was thinner in inferior sectors) [15]. Following this study, the performance of cpRNFL for distinguishing CON from GON has been evaluated by other studies with varying results [29, 30]. More recently, Andrade et al. reported that cpRNFL was significantly diminished in every sector in CON, except for the TS and TI [17]. In our study, we report a diffusely thinner cpRNFL in GON and CON compared to CG. Moreover, similar to the study aforementioned, although our results show that cpRNFL was thinner in N, T, NS e NI sectors in CON, and TI and TS sectors in GON, these differences were not statistically significant. Even though the differences between studies could be attributed to the different methodologies used, we can infer that while cpRNFL criteria may help differentiate CON from GON, additional parameters are needed to increase the diagnostic yield of OCT analysis.

Some reports suggest that macular thinning may precede cpRNFL and automated perimetry changes, although few studies use macular thickness parameters to distinguish between GON and CON [30–33]. Using the ETDRS grid, Lee et al. evaluated the damage induced by both diseases in cpRNFL, mRNFL, and mGCL [29]. The authors concluded that macular analysis of the IS and IN subfields (diminished in CON) in the mGCL was superior to the cpRNFL analysis in differentiating CON from GON. When distinguishing between both diseases, although we found significant differences in the IS sector in the IPL, the IN sector was consistently thinner in every layer of the GCC of CON patients. We believe the differences between studies are probably due to the GON sample characteristics, as we included patients with more advanced glaucomas [MD −6.01 (11.65) dB versu*s* −3.8 ± 4.8 dB] [29]. This is further confirmed by our comparison of GON with the healthy CG, in which we report a more diffuse macular damage in GON patients compared with the aforementioned study. We hypothesize that as GON progresses and macular damage becomes diffuse, the superior macular fibers may be targeted earlier than the nasal fibers, making nasal sectors a better parameter.

When evaluating all retinal layers, we observed that the neuroretinal degeneration in GON and CON was restricted to the GCC. Contrastingly, although not always significant, INL thickness was increased in most sectors of both diseases compared to CG. Recent studies have reported the same findings of increased INL thickness in GON and CON eyes and hypothesized that this retinal response might result from a defense mechanism of Müller glial cells to protect adjacent retinal ganglion cells against glaucomatous and retrograde degeneration retinal injury [21, 34–36].

Due to the inherent limitations of the ETDRS grid for optic neuropathies analysis (anatomically displaced in the vertical and horizontal meridians), we also performed a macular analysis of the GCC using a Garway-Heath-based grid. Similar to the ETDRS analysis, all three GCC layers were significantly affected in most sectors of both diseases compared to the CG. Furthermore, when CON versus GON is concerned, the macular nasal sectors were significantly more affected in CON in the mGCL and IPL, with the NI sector being the best parameter for differentiating between diseases. Moreover, macular thickness in CON was thinner than GON in almost every sector of the three layers of the GCC, except for the mGCL TI sector, which was thinner in GON, albeit not statistically significant. Furthermore, GON has been associated with an initial thinning of the TI and sparring of the TS sector until the later stages of the disease [21]. This finding is justified not only by the higher susceptibility of the inferior fibers to glaucomatous damage but also because the field of OCT macular analysis does not detect axonal damage in the ONH superior pole [37]. Yum et al. evaluated the performance of macular ganglion cell-inner plexiform layer (mGCIPL) thickness measurements in patients with NTG and patients with CON due to pituitary adenoma (with and without VF defects) using a similar classification [30]. Their results also show that the NI sector was the best discriminator for distinguishing between GON and CON and for early diagnosis of preperimetric CON versus healthy controls. Additionally, the TI sector was more diminished in GON patients than in CON patients. Differently from this study, we segmented the mGCL and IPL individually, which showed that the IPL sectors were better parameters for distinguishing CON from GON, than the mGCL sectors. A recent study that used macular evaluation to distinguish different stages of glaucoma found that mRNFL and mGCL were affected earlier than IPL and that the latter would eventually be damaged as GON progressed [21]. We hypothesize this is attributable to CON retrograde degeneration affecting the GCC diffusely while glaucomatous damage follows a progressive pattern. This finding is supported by the TI sector measurements that, contrarily to the mGCL, are more diminished in the IPL in CON patients. Finally, the mRNFL was the layer where we found fewer differences between diseases, possibly due to Garway-Heath-based grid optimization for mGCL and IPL over mRNFL analysis.

Due to the findings and GON and CON’s pathophysiology, we evaluated the diagnostic performance of the nasal/TI ratios in the three layers of the GCC. We observed that the NI/TI ratio, when applied to eyes with NI sectorial damage, presented the highest yield of diagnostic accuracy in the mGCL and IPL. This combination is crucial as applying this ratio alone in healthy CG may increase the rate of false positives. Moreover, combining NI thickness and NI/TI damage ratios in mGCL and IPL presented the highest accuracy for differentiating between CON and GON. Although our results are interesting, we suggest that macular evaluation of the nasal sectors and the NI/TI parameters should be combined with clinical and other OCT parameters (BMO-MRW/cpRNFL ratio) for referencing the patient for neuroimaging. Additionally, further studies evaluating the diagnostic yield of combining clinical and OCT parameters are paramount.

The strengths of our study are the presentation of macular parameters in the SD-OCT in evaluations that are objective, automated, and easy to interpret and use. Additionally, mGCL segmentation is already used in regular day-to-day glaucoma clinical practice. Another important point is that, unlike most studies evaluating macular damage, we used the ETDRS grid (to study all retina layers) and the Garway-Heath-based grid (more suited for optic neuropathies) to study the GCC specifically. Regarding methodology, we only included one eye per patient, thus avoiding the dependency and possible bias of bilateral enrollments. On the other hand, this limited our study by lowering our sample size. Another limitation of our study is the mRNFL analysis by the Garway-Heath-based grid. Although this analysis accurately distinguished CON and GON from healthy controls, it may not be as reliable for differentiating between diseases. Furthermore, the Glaucoma Module Premium Edition software requires good fixation during image acquisition which may not be possible in some patients. Additionally, even though myopia is a prevalent disease in GON, we excluded these patients from our study as it could have overestimated the retinal thinning compared to non-myopic patients. In the future, it would be interesting to analyze these same parameters in myopic patients and evaluate if macular thickness evaluation is a valuable criterion for distinguishing between GON and CON. Despite this, although this is a single-center study, we believe our sample is representative of the majority of the population, thus not affecting our external validity.

There is a paucity of literature concerning clinical and OCT findings that lead to an accurate diagnosis of GON and CON. Phenotyping these neuropathies is of utmost importance as it helps in their correct identification, which is both clinical and economically valuable as misdiagnosis carries significant morbidity. SD-OCT’s evaluation of the segmented macular layer damage may be a helpful add-on tool in the differential diagnosis between CON and GON when their manifestations overlap. NI and NS sector macular damage and the NI/TI ratio in mGCL and IPL may aid in the differential diagnosis of CON and GON.

## Supplementary information

ESM 1 (XLSX 16 kb)
